# Identifying advocacy strategies, challenges and opportunities for increasing domestic health policy and health systems research funding in Nigeria: Perspectives of researchers and policymakers

**DOI:** 10.1186/s12961-021-00701-5

**Published:** 2021-03-22

**Authors:** Ijeoma Nkem Okedo-Alex, Ifeyinwa Chizoba Akamike, Gladys Onyinye Olisaekee, Chinyere Cecilia Okeke, Chigozie Jesse Uneke

**Affiliations:** 1 African Institute for Health Policy and Health Systems, Ebonyi State University (EBSU), Abakaliki, Nigeria; 2 Department of Community Medicine, Alex Ekwueme Federal University Teaching Hospital, Abakaliki, Ebonyi State, Nigeria; 3 The Palladium Group, Ebonyi State Office, Abakaliki, Nigeria; 4 Department of Community Medicine, University of Nigeria, Nsukka, Nigeria

**Keywords:** Health policy and health systems research, HPSR, Domestic funding, Advocacy strategies, Perspectives, Researchers, Policymakers, Nigeria

## Abstract

**Introduction:**

Poor funding for Health Policy and Systems Research (HPSR) is a major constraint to the development, generation and uptake of HPSR evidence in Low and Middle-Income countries. The study assessed the status of HPSR domestic funding and advocacy strategies for improving HPSR funding in Nigeria. It equally explored the knowledge and perception of the domestic funding status of HPSR and the effect of capacity building on the knowledge of domestic funding for HPSR in Nigeria.

**Methods:**

This was a sub-national study involving policymakers and researchers from Enugu and Ebonyi States in Southeast Nigeria who participated in the sub-national Health Systems Global convening for the African region. A before-after study design (workshop) was utilized. Data collection employed semi-structured questionnaires, group and panel discussions. The workshop facilitated knowledge of HPSR, funding processes, and advocacy strategies for increased domestic funding for HPSR. Pre and immediate post-workshop knowledge assessments were done. Data were analyzed using SPSS version 25 and thematic analysis.

**Results:**

Twenty-six participants were involved in the study. Half were females (50.0%) and 46.2% were aged 35–44 years. Policymakers constituted 23.1% of the participants. Domestic funding for HPSR in Nigeria was adjudged to be grossly inadequate. Identified barriers to domestic funding of HPSR included bureaucratic bottlenecks, political and policy transitions, and corruption. Potential opportunities centered on existing policy documents and emerging private sector willingness to fund health research. Multi-stakeholder advocacy coalitions, continuous advocacy and researcher skill-building on advocacy with active private sector involvement were the strategies proffered by the participants. Pre-workshop, understanding of the meaning of HPSR had the highest mean ratings while knowledge of budgeting processes and use of legal action to enable opportunities for budget advocacy for HPSR funding had the lowest mean ratings. Following the capacity-building workshop, all knowledge and understanding parameters markedly improved (percentage increase of 12.5%–71.0%).

**Conclusion:**

This study found that there was paucity of domestic funding for HPSR in Nigeria alongside poor knowledge of budgeting and advocacy strategies among both policymakers and researchers. We recommend the deployment of these identified strategies and wider national and regional stakeholder engagement towards prioritizing and improving domestic funding for HPSR.

## Background

Research findings enhance evidence-based policy and decision-making at all levels of government and should help ensure that health interventions are targeted to significantly reduce a country's disease burden [1]. The complexity of health problems in Africa highlights the importance of strengthening national health research systems that generate timely knowledge and innovations to address local health challenges and advancement towards universal health coverage and health security [2]. However, failure to prioritize local research has resulted in a poor understanding of the determinants of poor health in Africa, and the unharnessed discovery of sound and contextualized interventions for addressing them. These social determinants are at the root of ill-health, health inequalities, and inequities [2, 3].

Over decades now, health systems including health research in sub-Saharan Africa have benefited massively from foreign aid [4–6]. While this has resulted in the improved health status of the populace, it has also been associated with increased dependence on external funding, pressure to meet the technical and administrative requirements of the funders, and most importantly the attendant focus on the priorities of funders rather than on national priorities. Several institutions conduct health research at the academic level and as government research agencies in Nigeria, but these institutions are often faced with serious challenges, notably gross underfunding and disconnect from national research priorities [1]. The absence of core funding for Health Policy and Systems Research (HPSR) has been recognized as a major constraint to the development of the field, generation, and uptake of evidence in Low and Middle-Income countries (LMICs) [7, 8].

A national health research agenda exists in Nigeria but lacks funding for its implementation as only 0.08% of the national health expenditure at the federal level is allocated to research. There is hardly any domestic funding for research at the sub-national level, with very poor private investment in health research and development [1]. In 2018, Nigeria committed to a 20% increase in budgetary support to health research institutions for research by 2022 as documented in the National Strategic Health Development Plan II (2018–2022) [1]. Despite this, domestic funding for HPSR in the country is inadequate and basically non-existent. Increasing domestic funding for HPSR has become progressively important given the double burden of disease, and the health system challenges in the country. Prioritizing funding for HPSR is vital to strengthening the health system and achieving sustainable development especially as HPSR has been steadily gaining grounds and growing capacities in research and academic institutions in Nigeria as in other LMICs [9, 10].

In order to achieve the national target for domestic funding for research, it is important that key stakeholders critical to the funding and conduct of research be identified, and the appropriate advocacy strategies used to stimulate the release of more funding for research. The purpose of this study was to assess the status of HPSR domestic funding and advocacy strategies for improving HPSR funding in Nigeria. It equally explored the knowledge and perception of HPSR domestic funding status and the effect of capacity building on the knowledge of domestic funding for HPSR in Nigeria.

## Methodology

### Study area

This research was carried out at the sub-national level with emphasis on two States namely Enugu and Ebonyi, both of which are located in the Southeast geopolitical zone of Nigeria. The Southeastern geopolitical zone consists of five States viz Anambra, Enugu, Ebonyi, Abia, and Imo States. Enugu and Ebonyi States are the leading States in health policy and systems research in Nigeria.

Enugu State is also known as the coal city State and has 17 Local Government Areas. It has about 12 tertiary academic institutions and 4 tertiary health institutions. The economy of the state is public sector driven, making civil service the predominant occupation of its working population. Other major occupations in Enugu are farming and trading. The majority of Enugu people are of the Igbo ethnic group and the predominant religion is Christianity.

Ebonyi State is the youngest State in the Southeastern part of Nigeria. There are thirteen Local Government Areas (LGAs) in the state. There are also 6 tertiary academic institutions and 2 tertiary health institutions in the State. About 75% of the Ebonyi population are involved in agriculture and the crops grown in the state include yam, rice, cassava, maize, and many others [11].

### Study participants

The participants in this study were drawn from both states and included career policymakers (e.g. permanent secretaries, directors, heads of departments, programme managers)[12, 13], political/elected policymakers from health and related areas, parliamentarians, global health practitioners, representatives of professional associations, Non-Governmental Organization (NGO) representatives and HPSR researchers.

### Study design and description

This was a before and after study embedded within the Health Systems Global (HSG) Africa regional network sub-national convening held on 4^th^ August 2020 in Abakaliki, Ebonyi State. This convening study was organized by the Africa HSG regional network based on the Sixth Global HSG Symposium on Health Systems Research (HSR 2020) sub-theme (Engaging political forces) and the HSG Africa’s priority area (advocating for increased domestic funding for research). A total of five convenings were held in the African region of HSG.

The study was held using a workshop design consisting of pre-workshop, workshop, and post-workshop phases. The pre and post-workshop assessments collected quantitative data while qualitative data was collected using panel and breakout sessions. All the participants were invited to the workshop by invitation letters which were sent 2 weeks before the event. This was followed up with text message reminders three days, and a day before the event. The intervention was the capacity-building sessions and they were focused on the use of HPSR evidence in policymaking, funding processes and state of HPSR funding in Nigeria, innovative platforms, and strategic opportunities for advocacy on increased domestic funding for HPSR in Nigeria.

### Sample size and selection of participants

A total of 26 purposively selected respondents participated in this study. This method of selecting policymakers and researchers for stakeholder events has been employed in previous studies.[13–15] The participants were selected based on the representativeness of the different stakeholders involved in HPSR in both States. The national guidelines on public gatherings not exceeding 30 persons in order to enforce physical distancing as part of the COVID-19 preventive measures influenced the sample size. Although the sample was limited to less than 30 persons, representatives of all the target stakeholder groups listed in the study participants’ subsection participated in the study.

### Data collection methods and tools

Both quantitative and qualitative data were collected. The study instruments included a questionnaire and topic guide for group and panel discussions.

### Quantitative data collection

A self-administered semi-structured questionnaire was used to collect information on knowledge and perception of respondents regarding HPSR funding. Semi-structured questionnaires have been used in previous studies among policymakers and researchers [16]. The questionnaire was made up of 3 sections. Section A was used to collect information on the socio-demographic characteristics of the participants. Section B was used to assess the adequacy of knowledge and understanding of HPSR, HPSR funding and advocacy for HPSR funding using a total of 15 questions on a 5-point Likert scale. Each question had five options on a rating scale of 1–5 points and scored as follows: 1 point = grossly inadequate; 2 points = inadequate; 3 points = fairly adequate; 4 points = adequate and 5 points = very adequate. The third section (section C) was used to assess the respondent’s perception of HPSR funding in Nigeria. Nine questions on a 5-point Likert scale were used to measure perception. Every single question had five options with the lowest point as 1 and the highest point as 5. It was scored as 1 point = strongly agree; 2 points = disagree; 3 points = indifferent; 4 points = agree and 5 points = strongly agree. The hard-copy questionnaires were completed before and after the capacity building sessions. However, only the knowledge section was re-assessed after the capacity building sessions as the post-workshop assessment.

### Qualitative data collection

#### Group discussions

A total of four group discussions were conducted. Two of the groups were focused on stakeholder mapping and power analysis while the other two groups discussed the strengths, weaknesses, opportunities, and threats (SWOT) analysis of domestic funding for HPSR in Nigeria. Writing materials (plain and cardboard sheets) and simplified, easy to understand templates for SWOT analyses (SWOT analysis box) and stakeholder power analysis (matrix and tables) were provided for the group work. Overall, the group discussions were used to better understand the importance of domestic funding to HPSR, sources of domestic funding, challenges, and consequences of lack of domestic funding for HPSR in Nigeria and advocacy strategies to improve domestic funding for HPSR in Nigeria.

The group discussion guide had 5–6 questions with probes and there were about 6–8 discussants per group. Each group discussion lasted about 35 min. To enable a participatory approach, ensure peer support, and minimize external interferences from the researchers, each group appointed a note-taker who wrote down salient points from the group discussions. Afterward, this was presented using flip charts by the group representative and critiqued by the other participants. An independent non-participatory note-taker was assigned to each group to write the responses of the participants.

#### Panel discussions

The panel discussion was made up of about 6 panelists (commissioner for Donors and Grants, permanent secretaries and directors in the ministry of health, NGO representatives, HPSR researcher, and parliamentarian) and a moderator who anchored the discussions in a flexible manner. The themes discussed were: current state of health research and HPSR funding in Nigeria; research funding processes; influencing the budget-making process to increase HPSR funding and strategies for increasing domestic funding for HPSR in Nigeria. The panel discussion lasted for about one hour. Notes were taken by three note-takers to ensure that all the responses of the panelists were captured.

### Capacity building session/intervention

The two capacity-building sessions were facilitated by a member of the research team who was skilled in HPSR and stakeholder engagement for use of evidence in policymaking. The topics covered were: a) overview of HPSR, the Nigerian health system, and health research funding b) identifying stakeholders and advocacy strategies for increased domestic funding for HPSR in Nigeria. PowerPoint presentations were used for the teaching sessions. All lectures were delivered in simplified, practical, and easily comprehensible patterns. Complex mathematical or scientific computations/models were avoided for the benefit of non-specialists who participated in the workshop. The participants were also provided with writing materials such as jotters, plain sheets, and pens. Questions and feedbacks were entertained at the end of each session. Each session lasted an average of one hour. The entire convening lasted from 10 a.m-3 p.m.

### Post-workshop/intervention survey

At the end of the capacity building sessions, the post-workshop assessment questionnaire was administered to the participants. The same questionnaire used at the pre-workshop survey was the same one used for post-workshop assessment. The aim of the post-workshop assessment was to evaluate the impact of the workshop on level of knowledge and understanding of respondents regarding HPSR funding in Nigeria.

### Data analysis and management

#### Measurement of variables

The independent variables include socio-demographic and work characteristics such as: age, gender, organization, and designation/position.

The dependent variables were the knowledge and perception of HPSR funding in Nigeria.

To grade the knowledge and perception of HPSR funding, the Mean Neutral Rating (MNR) of the Likert scale responses was done using the methods developed at McMaster University Canada by Johnson and Lavis [17]. For knowledge and understanding of HPSR funding, mean knowledge scores between 3.00 and 5.00 were categorized as good while values less than 3.00 were taken as poor knowledge. Mean perception scores between 3.00 and 5.00 were categorized as good while values less than 3.00 were taken as poor.

#### Data analysis

Quantitative data analysis was done using SPSS version 25. Frequencies, proportions, means, and standard deviations were computed. Results were presented using frequency tables.

Qualitative data analysis commenced with a review of hand-written notes from the group discussions to confirm the completeness of the information documented. Pre-conceived themes were generated from the discussion guide to develop a coding framework. The notes were then read to achieve familiarization and identify any themes that were not in the coding framework. The themes included in the final coding framework are as follows: (i) the importance of domestic HPSR funding, (ii) sources of HPSR funding, (iii) consequences of lack of domestic funding for HPSR, (iv) strategic stakeholders for increasing HPSR funding (v) strategies to improve domestic funding for HPSR in Nigeria, (v) strengths, weaknesses, opportunities, and threats to HPSR funding in Nigeria.

## Results

Twenty-six participants were involved in the study. Half of the participants were females (50.0%) and 46.2% were aged 35–44 years. Policymakers constituted 23.1% of the participants while 65.4% had spent < 5 years in their designated position (Table 1).

**Table 1:** Socio demographic characteristics of subnational policymakers and researchers in the Nigerian sub-national convening (*n* = 26)

Variable	Frequency	Percent (%)
Age		
25–34	4	15.4
35–44	12	46.2
> 45	10	38.5
Gender		
Male	13	50
Female	13	50
Participant type		
Researcher	18	69.2
Policy maker	6	23.1
Non-governmental organization	2	7.7
Duration in designation/position		
< 5	17	65.4
5–10	5	19.2
11–15	3	11.5
> 16	1	3.8

### Qualitative results

#### Panel discussions

Inadequate domestic HPSR funding.

The discussants all affirmed that domestic funding for HPSR was not adequate however it should be prioritized in order to manage what is available. Some of the supporting quotes are shown below:

“Funding for health can never be adequate. The question should be how we should make the available resources enough?” (Panelist 1)

“Funding is inadequate, there’s a problem with prioritization. Nobody takes health research funding as a priority” (Panelist 2)

Factors that affect domestic funding for HPSR

Some factors that affect funding as highlighted by the discussants include: political agenda and interests, poor management, and influence of external funders. Some of the supporting quotes include:

“Most leaders are interested in scoring political points thus physically visible projects like roads and buildings are prioritized over research” (Panelist 1)

“External funders have their own stereotyped agenda/programme and because they significantly fund most programmes, they decide the researches to be conducted” (Panelist 3)

Budgeting for general health research and HPSR

The discussants affirmed that HPSR is not well represented in the budget and also buttressed the fact that the proportion of national budget allocated to research generally is not being implemented. Furthermore, some level of politics has been observed in the disbursing of funds for research at the tertiary education level. They suggested the need to institutionalize health policy and systems research in the ministry of health and also build the capacity of those involved in the budgeting process. Some verbatim responses are below:

“2% of the national health budget is meant to be for research but this is not implemented” (Panelist 4)

“Usual practice is to copy and paste the old budget. No real needs assessment. There’s need to build capacity of those that prepare the budget and carry them along” (Panelist 2)

Advocacy strategies to improve domestic funding for research

Discussants proffered various advocacy strategies that may be of potential benefit in improving domestic funding for research. These include capacity building for leaders, decision-makers, and citizens, proper budgeting process, financial autonomy for departments, and formation of coalitions for advocacy by professional bodies, lobbying, among others. Some supporting quotes are below:

“Director Generals of certain institutions should be approached and bought in. These can then join in advocating. Also, professional bodies such as Nigerian Medical Association (NMA), National Asoociation of Nigeria Nurses and Midwives (NANM), Pharmacy associations, etc; legal institutions; pharmaceutical research institutes should be engaged and carried along” (Panelist 1)

*“The departments of planning, research and statistics exist but the ‘R’ is missing so there should be capacity building in three ways: planning, research and statistics, for decision makers in getting evidence into policymaking and for citizens to be able to make demands for budget implementation and to have a voice in budget implementation” (Panelist 4)*

*“Lobbying is necessary. The people in the health sector need to begin to shout and write to ensure that funding is channeled to HPSR. We need to put the leaders under pressure” (Panelist 6)*

Regarding the stereotyped nature of donor programmes and funding, participants recommended domestic ownership of the health programmes to ensure sustainability. They also deemed it necessary to properly integrate the government programmes with that of the donors. Below are supporting quotes:

“When partners arrive, the Government should own the programmes. Something is already being done to integrate Government and donors for better sustainability” (Panelist 1)

### Group discussions

The importance of domestic funding for research included the conduct of policy-relevant research, enhanced institutional visibility and sustainability of programmes, improved trust, and ownership of research evidence for policymaking and enhanced community ownership and participation. Both public and private sector sources were identified for domestic funding of HPSR (Table 2).

**Table 2:** Summary of themes from the group discussions among subnational policymakers and researchers in the Nigerian sub-national convening

Theme	Outcome of group discussion
(A). Importance of domestic funding for HPSR	
(i). To the researcher	Better researcher-funder relationship Improves access to funding Conduct of policy-relevant research
(ii). To institution	Increases research in priority areas Enhanced institutional visibility and sustainability of programmes Improves human research development
(iii). To government	Improves trust and ownership of research evidence Increases researcher-policymaker communication Avoids parallel funding Institutionalizes evidence informed policymaking
(iv). To society	Improves societal trust in research findings Enhances community ownership and participation Encourages need-based research
(B). Potential and current Sources of funding for HPSR	
(i). Public	Real Sector Intervention fund, general tax revenues, value added tax, internally generated revenue
(ii). Private	Corporate organizations like commercial banks, Non-governmental organizations and donor agencies, philanthropists, faith-based organizations
(C). SWOT Analysis of domestic funding in Nigeria	
(i). Strengths	Existing national priority areas, existing researcher interests
(ii). Weaknesses	Corruption, budget approval not being equivalent to release of funds, bureaucratic bottlenecks, tribalism, poor needs assessment
(iii). Opportunities	Already existing budget allocation for health research, corporate agencies and companies that have shown interest in funding healthcare research
(iv). Threats	Bureaucracies/ bottlenecks limit release of budget allocation, non-sustainability of funding policies due to political changes in government regimes, prevailing poor accountability resulting in corrupt funding practices, vested interests, nepotism
(D). Consequences of poor domestic funding	
(i). To the researcher	Low morale for research and lack of fulfillment, poor career development, low visibility for researchers
(ii). To institution	Poor institutional research development and visibility, limited data for advocacy, limited priority-based research, poor sustainability of programmes, poor institutional contribution to national development
(iii). To government	Donor-dependent research to the detriment of national research priorities, poor linkages between researchers and policymaking resulting in poor use of evidence in policymaking, poor sustainability of policies and projects
(E). Strategies to improve domestic funding for HPSR	
(i). To the researcher	Researcher capacity enhancement to improve advocacy skills, advocacy coalitions by researchers and professional associations for demand creation on research funding, improved quality and alignment of research with national health research priorities
(ii). To institution	Enhanced researcher capacity at institutional level Institutional advocacy for research funding
(iii). To government	Budgetary advocacy to ensure inclusion of HPSR funding in the national and subnational budgets, stakeholder advocacy and lobbying to ensure that approved fund is released, media engagement and networking, demanding for accountability

The strengths and opportunities for domestic HPSR funding in Nigeria were existing researcher interests and policy documents on research priorities and budget allocation to health research. The weaknesses and threats were bureaucratic bottlenecks at budget allocation, approval and fund release, transitory political leadership, poor accountability and corruption (Table 2).

At the researcher level, low morale for research, and poor career development were the consequences of poor funding while limited data for advocacy and low visibility were the institutional consequences. At the governmental level, continued donor-dependence, non-priority research, and non-sustainable programmes were highlighted as consequences. The strategies recommended for increased domestic funding of HPSR were researcher capacity enhancement to improve advocacy skills, advocacy coalitions by researchers, and continual stakeholder lobbying (Table 2).

### Quantitative results

The mean rated perception of HPSR funding in Nigeria was lowest with respect to sole funding of health policy and systems research by the Government (1.96) and adequacy of funds released for health policy and systems research in Nigeria (2.31). The statements (parameters) with the highest mean perception ratings were that domestic funding of health policy and systems research in Nigeria will remove or at least reduce dependence on external sources of health research funding (4.31) and that domestic funding of health research in Nigeria will improve the focus of health policy and systems research to meet national research priorities and subsequently health outcomes for the population (4.27) (Table 3).

**Table 3:** Perception of HPSR funding in Nigeria among subnational policymakers and researchers in the Nigerian sub-national convening (*n* = 26)

Parameter assessed	Mean
Funding for health policy and systems research in Nigeria should be sourced from within the country	4.15
Adequate funds are earmarked by government for health policy and systems research in Nigeria	2.46
Adequate funds are released for health policy and systems research in Nigeria	2.31
Nigeria has the capacity (human, financial and technical ability) to fund health policy and systems research in the country	4.04
Domestic funding of health policy and systems research in Nigeria will remove or at least reduce dependence on external sources of health research funding	4.31
Domestic funding of health research in Nigeria will improve focus of health policy and systems research to meet national research priorities and subsequently health outcomes for the population	4.27
All domestic funding for health policy and systems research -should be from the Government alone	1.96
Different stakeholders (Private organizations, CSOs, Professional bodies, Media etc.) have been engaged and are aware of the need to fund health policy and systems research in Nigeria	2.46
If different stakeholders (Private organizations, CSOs, Professional bodies, Media etc.) are engaged, they will be willing to fund HPSR in Nigeria	4.03

Before the capacity building, understanding of the meaning of HPSR had the highest mean rating of 4.15 while knowledge of how to use legal action to enable opportunities for budget advocacy for increased funding of HPSR had the lowest mean rating of 2.42. After the capacity building exercise, all parameters assessed had a mean greater than 4.00 and the lowest range was 3–5 (Fairly adequate to very adequate knowledge). Understanding of the meaning of health policy & systems research has the highest mean rating of 4.67 (Table 4).

**Table 4:** Comparison of the Pre-and Post-workshop knowledge and understanding of HPSR, HPSR funding, advocacy and stakeholder engagement among subnational policymakers and researchers in Nigeria

Parameter assessed	PRE mean	POST mean	Mean increase	Percentage mean increase
Understanding of the meaning of health policy and systems research	4.15	4.67	0.52	12.53
Knowledge about the WHO ranking of the performance of the Nigeria health Systems	3.38	4.48	1.1	32.54
Understanding of why government policies are not resulting in the expected impact	3.85	4.57	0.72	18.70
Understanding of the complexity of the policymaking process	3.73	4.52	0.79	21.17
Knowledge and understanding of the key characteristics of health policy & systems research	3.73	4.38	0.65	17.42
Knowledge and understanding of evidence-informed policymaking, systems thinking, policy cycle and Knowledge-To-Action Cycle in health policymaking	3.73	4.57	0.84	22.52
Knowledge and understanding of Budget Advocacy Skills, including analytical skills, communication skills, and collaboration/ interpersonal skills for increasing funding of HPSR	3.27	4.62	1.35	41.28
Knowledge and understanding of budget advocacy tools, and tactics including building a constituency, generating media coverage, and lobbying for increasing funding of HPSR	3.04	4.29	1.25	41.11
Knowledge and understanding of budget advocacy analysis including accuracy, accessibility and timeliness for increasing funding of HPSR	2.88	4.14	1.26	43.75
Knowledge and understanding of how to engage Stakeholders and Legislature in advocacy for increasing funding of HPSR	3.23	4.43	1.2	37.15
Knowledge and understanding of the Political and Policy Context of advocacy for increasing funding of HPSR	3.00	4.52	1.52	50.67
Knowledge and understanding of building alliances and coalitions for your budget advocacy for increasing funding of HPSR	2.92	4.43	1.51	51.71
Knowledge and understanding of how to use legal action to enable opportunities for budget advocacy for increasing funding of HPSR	2.42	4.14	1.72	71.07
Knowledge about how HPSR is funded in Nigeria	2.81	4.29	1.48	52.66
Knowledge about domestic sources of funding for HPSR in Nigeria	3.00	4.48	1.48	49.33

The parameters with the highest mean increase in knowledge post-intervention was understanding how to use legal actions to enable opportunities (1.72, 71.07%) and understanding of the political and policy context of advocacy (1.52, 50.0%). Understanding of the meaning of HPSR had the lowest mean increase of 0.52 (Table 4).

## Discussion

The study assessed the status of HPSR domestic funding and advocacy strategies for improving HPSR funding in Nigeria. It equally explored the knowledge and perception of HPSR domestic funding status and the effect of capacity building on the knowledge of domestic funding for HPSR in Nigeria.

Sound understanding of both potential and current funding sources to support HPSR activities is important for informing advocacy efforts to relevant stakeholders [8]. Inadequacy of domestic funding and minimal engagement of multi-stakeholders (especially private sector) towards funding HPSR in Nigeria was well highlighted quantitatively and qualitatively. The participants identified the importance of domestic funding for HPSR across researcher, institutional, governmental, and societal levels. Overall, the major advantages of domestic HPSR funding mentioned were the improved conduct of policy-relevant research, enhanced institutional visibility and sustainability of programmes, trust and ownership of research evidence, and eventual use of HPSR evidence in policymaking. Ownership of research evidence not only spurs uptake into policy and practice but also provides concrete links between research and decision-making [18].

It was interesting to note that participants considered private sector involvement important in enhancing domestic funding for HPSR. Notably mentioned private sector actors were non-governmental organizations, philanthropists, and cooperate bodies like banks. In addition, donor agencies were also distinctively mentioned possibly due to the fixation and dependence on donors for funding HPSR as has been the norm. There is an implicit concern that a shift in key donor priorities towards HPSR funding could have catastrophic consequences given the relatively low funding base of HPSR [8]. The conduct of relevant priority-based donor-funded health research can be strengthened with local actor involvement in the design, implementation and evaluation of such research projects [19]. In line with decolonizing global health, it is necessary that policymakers and researchers in donor-dependent countries like Nigeria begin to re-orient their mindset towards funding independence and also demand for local funding [20].

Bureaucratic bottlenecks such as non-release of allocated funds for health research, corruption/poor accountability and political determinants such as unstable government regimes undermine the actualization of domestic HPSR funding in Nigeria. Political stakeholders and external funders could have varying priorities which may not include funding HPSR. However, some windows of opportunity exist such as existing national policies stating research priorities and proportion of funding to be allotted to health research[1], growing community of HPSR researchers, and potential private sector interests/willingness to fund HPSR in Nigeria. These need to be harnessed in addition to addressing the identified barriers to domestic funding. Research priorities also need to go beyond biomedical research to HPSR in order to strengthen the health system.

The participants posited that insufficient funding could have dire consequences on the health system and evidence-informed policy making ranging from low researcher morale, limited priority-based research, weak researcher-policymaker link, perpetual reliance on donors, and non-sustainability of policies/programmes. Such burgeoning consequences of poor funding of research generally and HPSR, in particular, have been previously identified and represent a loud call for action. Some actionable strategies were highlighted to averting these impending problems were provided by the participants. These include researcher and institutional capacity enhancement for research and advocacy, advocacy coalitions, and continuous advocacy to stakeholders including the use of media. Improving researcher capacity for HPSR has been previously recommended [7, 8], however there has been little or no focus on building researcher competencies for advocacy. Given that researchers require funding to conduct quality research, more focus should be placed on developing their skill for advocacy to funders especially in the domestic setting.

At the pre-workshop stage, understanding of HPSR and policymaking process, had higher mean scores while understanding of the budgeting process and advocacy mechanisms for increasing HPSR funding had lower mean scores. The low knowledge level could stem from the fact that funding for HPSR has been largely donor-driven [4], without the active involvement of both researchers and policymakers in budget allocation and other budgeting processes and structures. More so, participants also identified that domestic funding for HPSR was near to non-existent thus not affording platforms for the local HPSR community to be involved in the funding process. The findings highlight the need for stakeholders from both policy making and researcher angles to properly understand the critical steps in funding in order to be able to advocate appropriately for HPSR funding. Participants perceived human resource capacity for conducting HPSR in Nigeria to be satisfactory as reflected by the high mean scores. Studies have shown that HPSR capacity in Nigeria has steadily developed over the years [10, 21]. hence more national and sub-national level support is required to maximize the potentials therein towards strengthening the weak Nigerian health system. At the regional level, the West African Health Organization (WAHO) has significantly contributed to strengthening National Health Research Systems and research partnerships [22]. Following the capacity building workshop, all knowledge and understanding parameters markedly improved (percentage increase of 12.5%–71.0%). This could be because this face to face workshop brought participants away from everyday distractions (work and personal), encouraged active participation, and gave the opportunity for feedbacks. Workshops have been proven to be effective in improving capacity of both policymakers and researchers at both individual and institutional levels [13, 15, 23–26].

This study had a few limitations. Firstly, the findings of this study were based on self-reports and could be prone to social desirability bias because of its subjective nature. However, participants were encouraged to give sincere responses and were assured of the confidentiality and anonymity of their responses. Only two States out of the thirty-six States in Nigeria were involved in the study thus limiting the generalizability of the results from this study. Nonetheless, the findings could be applicable to other sub-national levels in Nigeria given the similarities in governance structure across States in the country.

Some strengths of the study are as follows: To the best of our knowledge and search, this is one of the few studies that have explored the status of domestic funding for HPSR. This study also involved a wide range of stakeholders and utilized a mixed-methods design for data collection.

### Implications of research findings on policy and practice

Grossly inadequate domestic funding for HPSR will negatively impact quality evidence generation and use of context-specific and relevant evidence for policy-making, programming and practice. Thus, this study’s findings represent an urgent call for policy development, implementation, and evaluation geared towards institutionalizing the provision of funds for the generation of research evidence towards strengthening health systems. Our findings also highlight the need to build the capacities of policymakers, and HPSR researchers on budgeting and advocacy strategies for improved HPSR funding. The workshop-based capacity-building strategy as used in this study is highly recommended towards developing a critical mass of researchers and policymakers who are well-versed in budgeting and advocacy for HPSR funding. The advocacy strategies isolated in this study at governmental, institutional, researcher and private-sector levels are starting points for active and meaningful multi-stakeholder engagement towards improving domestic funding for HPSR.

### Areas for future research

The areas for future research will involve identifying and deploying cost-effective, context-specific strategies and interventions to address the known barriers to domestic funding for HPSR. These will need to be tailored to the different stakeholder groups, interests, and roles and should not preclude strengthening the already existing and recognized mechanisms that support domestic funding for HPSR. Interventions that employ the advocacy strategies suggested in this study need to be evaluated for effectiveness and impact on domestic funding for HPSR. It is also important to assess the intermediate and long-term effects of capacity-building on HPSR for both researchers and policymakers.

## Conclusion

This study found that there was paucity of domestic funding for HPSR in Nigeria alongside poor knowledge of budgeting and advocacy strategies among both policymakers and researchers. Some of the strategies for increasing domestic funding of HPSR identified include the formation of advocacy coalitions, researcher skill-building on advocacy for funding and continuous advocacy to both public and private sector stakeholders. The study also showed that capacity building improved the knowledge and understanding of HPSR concepts, funding processes, and advocacy mechanisms for improving HPSR funding. We recommend the deployment of these strategies and wider national and regional stakeholder engagement towards prioritizing and improving domestic funding for HPSR.

## Data Availability

The datasets used and/or analyzed during the current study are available from the corresponding author on reasonable request.

## Abbreviations

LMIC: Low and Middle Income Countries
HPSR: Health policy and systems research
SD: Standard deviation
SPSS: Statistical Package for |Social sciences
